# Around the Hospitals

**Published:** 1892-07-02

**Authors:** 


### Around the Hospitals.

**The Royal Hospital for Women.—Floral Fete at the Mansion House.**—The funds of this hospital should be considerably augmented by the very large crowd which poured into the Mansion House on the 23rd inst., though it is to be feared that the popularity of the fête rather defeated its own ends. It was known that upwards of 6,000 tickets were purchased, but owing to a delay in opening the doors, a surging crowd of ladies and children who wished to see the opening ceremony, poured in and caused such a block that locomotion was impossible, and but few, comparatively, could get near to the flower stalls, and those who did buy were scarcely able to remove their purchases, which suffered a great deal in the scramble. The heat, too, was so intense that the flowers, especially roses, drooped and lost much of their beauty rather early in the afternoon. The stalls were arranged as a series of bowers, of which the most prominent was that of the Lady Mayoress, in the centre of the Egyptian Hall. She was assisted by her daughters, and by Lady Edward Somerset, Lady Arthur Hill, Lady Hanson, and a dozen more young ladies, whose costumes rivalled the flowers in beauty and variety. Mrs. Foster's stall was conspicuous on account of its very elaborate decorations. Above it hung a civic crown made entirely of roses, and it was linked by garlands of roses to pillars of ivy. The "children's stall" was quaint with its collection of "olde Englyshe floweres." Mrs. Brinley Richards' sedan chair baskets, heaped with roses, were very attractive; and Mrs. Frank Grimwood (of Manipur) did a brisk business in roses, sent by the Princess of Wales from Sandringham, and bearing labels to that effect. Connoisseurs were inclined to award the palm to Lady Alexander Kennedy for her wonderful roses "all from Scotland"; one bouquet, Souvenir d'un Ami, is unforgetable. Mrs. Soulsby's stall had a splendid collection of Mr. Alfred de Rothschild's famous carnations. Mrs. Eastwood's fruit and orchids were a marvel. The beautiful Mrs. Weguelin had no difficulty in disposing of two sprays of Odontoglossum vexillarium at five guineas each. Mrs. Staples had imported a number of baskets from Madeira, and the effect of these when filled with cattleyas was charming. Mrs. Faudel Phillips had also a fine show of orchids, and a row of baskets of exquisite lilies further embellished the front of her stall. Mrs. Sutherland and Mrs. Wardrop had an original scheme of colour, for they had kept chiefly to Iceland poppies and blue hydrangeas. Lady Bective's punch bowls full of roses against a dark background made an excellent effect. The Miss Faudel Phillips presided over the strawberries and cream, which they ladled from enormous gold vases of appropriate civic splendour. Strawberries are now so plentiful that many might have hesitated to risk the endeavour to eat in such a crowd; but those golden vases imparted a fairy-tale flavour—Dick Whittington, Gog and Magog, and the lovely Princesses who are united to heroic Princes and "live happy ever after and eat strawberries and cream out of golden vases every day." The sale was opened by Princess Christian, who was accompanied by her daughter, of whom everyone struggled to get a glimpse on account of the rumour that her engagement is immediately to be announced. Princess Christian had modified her mourning slightly for the occasion, and wore a white broché vest embroidered with gold to lighten up her dress of black and jet, and her bonnet was black lace with a wreath of white roses. She was preceded by the City Marshall and Swordbearer, and the Lord Mayor conducted her to the Lady Mayoress's stall. The Lord Mayor then made a brief speech, thanking her for her presence, and saying how warmly she always gave her support to works of charity, especially in connection with women and children. The Princess replied that it gave her very great pleasure to declare the sale open. She then endeavoured to make a tour of the stalls, but, owing to the crowd, this was almost out of the question; people were wedged in shoulder to shoulder, and could neither move backwards or forwards.

